# Vascular and pulmonary effects of ibuprofen on neonatal lung development

**DOI:** 10.1186/s12931-023-02342-4

**Published:** 2023-02-02

**Authors:** Xueyu Chen, Dongshan Han, Xuan Wang, Xuemei Huang, Zilu Huang, Yijun Liu, Junyan Zhong, Frans J. Walther, Chuanzhong Yang, Gerry T. M. Wagenaar

**Affiliations:** 1 Laboratory of Neonatology, Department of Neonatology, Affiliated Shenzhen Maternity and Child Healthcare Hospital, The First School of Clinical Medicine, Southern Medical University, Shenzhen, China; 2 Department of Pediatrics, David Geffen School of Medicine, University of California Los Angeles, Los Angeles, CA USA; 3 Lundquist Institute for Biomedical Innovation at Harbor-UCLA Medical Center, Torrance, CA USA; 4 Faculty of Science, VU University Amsterdam, Amsterdam, The Netherlands

**Keywords:** Bronchopulmonary dysplasia, Lung inflammation, Alveolarization, Pulmonary arterial hypertension, Angiogenesis, Cell cycle arrest, Nonsteroidal anti-inflammatory drug

## Abstract

**Background:**

Ibuprofen is a nonsteroidal anti-inflammatory drug that is commonly used to stimulate closure of a patent ductus arteriosus (PDA) in very premature infants and may lead to aberrant neonatal lung development and bronchopulmonary dysplasia (BPD).

**Methods:**

We investigated the effect of ibuprofen on angiogenesis in human umbilical cord vein endothelial cells (HUVECs) and the therapeutic potential of daily treatment with 50 mg/kg of ibuprofen injected subcutaneously in neonatal Wistar rat pups with severe hyperoxia-induced experimental BPD. Parameters investigated included growth, survival, lung histopathology and mRNA expression.

**Results:**

Ibuprofen inhibited angiogenesis in HUVECs, as shown by reduced tube formation, migration and cell proliferation via inhibition of the cell cycle S-phase and promotion of apoptosis. Treatment of newborn rat pups with ibuprofen reduced pulmonary vessel density in the developing lung, but also attenuated experimental BPD by reducing lung inflammation, alveolar enlargement, alveolar septum thickness and small arteriolar wall thickening.

**Conclusions:**

In conclusion, ibuprofen has dual effects on lung development: adverse effects on angiogenesis and beneficial effects on alveolarization and inflammation. Therefore, extrapolation of the beneficial effects of ibuprofen to premature infants with BPD should be done with extreme caution.

## Background

Major advances in neonatal intensive care have not reduced the incidence of bronchopulmonary dysplasia (BPD) or neonatal chronic lung disease (CLD) in premature infants, because increased neonatal survival has shifted the affected population to premature infants born at less than 28 weeks of gestation [1, 2]. The incidence of BPD is stable at 35–40% of extremely premature infants [2, 3]. Treatment of respiratory failure due to lung immaturity and surfactant deficiency in these extremely premature infants with invasive respiratory support and supplemental oxygen may injure the developing lung permanently [4]. BPD is characterized by a reduced alveolar surface and impaired lung function due to enlarged alveoli caused by oxidative stress-induced lung damage and arrested alveolar development [1]. Prenatal insults, perinatal inflammation, oxidative stress and pulmonary arterial hypertension (PAH) complicate BPD pathogenesis and contribute to adult lung disease, like COPD, at relatively young ages [2, 3, 5, 6]. Effective pharmacological treatment for BPD is lacking and badly needed.

The neonatal rat is a suitable animal model for studying BPD pathogenesis and novel treatment options [7–10]. These rodents are born during the saccular stage of lung development, mimicking the lung development stage of infants at high risk for BPD, and develop chronic lung inflammation, followed by persistent alveolar simplification, lung fibrosis, PAH and right ventricular hypertrophy (RVH) after exposure to hyperoxia [1, 11].

Ibuprofen is a potent nonsteroidal anti-inflammatory drug (NSAID) that is extensively used for the treatment of colorectal cancer, lung inflammation in cystic fibrosis, and closure of a patent ductus arteriosus (PDA) in premature neonates [12–15]. However, information about its effect on aberrant lung development after premature birth and the pathogenesis of BPD is incomplete and controversial, ranging from concerns about adverse effects, no impact, to beneficial effects on BPD in premature infants [15–20]. Our previous clinical study and a meta-analysis have indicated an increased risk for BPD in ibuprofen-treated infants [16, 20]. Other experimental studies suggested an anti-angiogenic effect of ibuprofen in ocular angiogenesis in neonatal rats [21] and embryonic development in zebrafish [22]. Considering the essential role of angiogenesis in the pathogenesis of BPD [2] and the fact that each year millions of premature infants receive ibuprofen for PDA closure [17], of which some are exposed to repeated or prolonged courses of ibuprofen treatment [23], there is an urgent need to unravel the potential role of ibuprofen in normal lung development and BPD pathogenesis after premature birth.

To advance our knowledge on the effect of ibuprofen treatment on perinatal lung development and BPD, we studied the effect of ibuprofen on endothelial cell function in cultured human umbilical vein endothelial cells (HUVECs), and the effect on alveolar and vascular development and lung inflammation in neonatal rats kept under conditions of normoxia or hyperoxia to induce experimental BPD [24].

## Materials and methods

### In vitro studies

#### Human umbilical vein endothelial cells (HUVECs)

HUVECs were isolated from the umbilical cord, as previously reported [25]. Briefly, an umbilical cord of 10–20 cm in length was collected after obtaining consent from the parents and processed in a biohazard cabinet. The umbilical vein was cannulated and rinsed using sterile 0.09% saline to remove blood. One millilitre of 0.2% collagenase (C0103, Sigma-Aldrich, St. Louis, MO, USA) was injected into the umbilical vein and incubated for 10 min at 37 °C. The umbilical cord was gently squeezed to facilitate the detachment of endothelial cells. Hereafter, the umbilical vein was rinsed with endothelial cell medium (ECM, 1001, ScienCell, Carlsbad, CA, USA), containing 10% fetal bovine serum (FBS, SV30208, HyClone, Marlborough, MA, USA) and Pen/Strep (#15140-122, Gibco, Fremont, CA, USA) to harvest the cells. HUVECs were collected by centrifugation at 750×*g* for 10 min, resuspended in medium and cultured at 37 °C in 95% air/5% CO_2_ humidified with water. Cells within passage 3–7 were used for the experiments.

#### Tube formation assay

96-well plates were pre-cooled and coated with 50 µL of Matrigel basement membrane matrix (#354234, Corning, NY, USA) per well. The plates were incubated at 37 °C for 1 h to allow the basement to polymerize. After the gel became solid, 1 × 10^4^ HUVECs were seeded with 100 µL of complete ECM containing DMSO (0.1%, v/v) or 100 µM, 500 µM or 1000 µM of ibuprofen. Plates were incubated at 37 °C in 95% air/5% CO_2_ for 6 h. Tube formation of HUVECs under different conditions was photographed with a camera mounted on a microscope (IX73, Olympus, Tokyo, Japan) and analyzed with Image J software (NIH, USA). Five technical repeats were performed.

#### Wound healing assay

HUVECs were seeded into a 12-well-plate at 2 × 10^5^ cells/well in ECM supplemented with 10% FBS and 1% Pen/Strep. The cells reached a confluency of 80–90% after 24 h of incubation. Then, the culture medium was removed, monolayers were scratched using a 200 µL pipette tip to make a straight wound. The wound was rinsed twice with phosphate-buffered saline (PBS) and incubated with ECM free of FBS and treated with DMSO (0.1%, v/v), 100 µM, 500 µM and 1000 µM of ibuprofen dissolved in DMSO for 24 h at 37 °C in 95% air/5% CO_2_. The healing process was monitored under a microscope. The wounds were photographed at 0, 12 and 24 h after the scratch and analyzed with Image J software. At least 5 pictures of each well in three technical repeats were analyzed.

#### Immunofluorescence

HUVECs treated with different concentrations of ibuprofen were fixed with 4% paraformaldehyde and permeabilized with 0.5% Triton X-100. The cells were then incubated overnight at 4 °C with Ki67 antibody (ab16667, Abcam, Fremont, CA, USA; diluted 1: 250), DNA damage marker, 8-hydroxy-2'-deoxyguanosine (8-OHdG, 12501, QED Bioscience, San Diego, CA, USA; diluted 1:1000), or 1% BSA (A1933, Sigma-Aldrich, St. Louis, MO, USA) as a control, followed by incubation with Alexa Fluor 488/555-conjugated donkey anti-rabbit/mice antibody (A-21206 and A21422, Invitrogen, Waltham, MA, USA, diluted in 1:1000) for 2 h in the dark. The cells were covered with Prolong Gold antifade reagent with DAPI (8961, Cell Signaling Technology, Danvers, MA, USA) and incubated in the dark for 24 h and then sealed with nail polish. The cells were studied with an Olympus IX73 microscope (Tokyo, Japan), equipped with a camera. Pictures were taken with a consistent exposure time, and analyzed with Image J software. At least 6 pictures of each well in three (Ki67) or five (8-OHdG) technical repeats were analyzed.

Paraffin-embedded rat lung tissue sections were incubated with an antibody against vWF (A0082, Dako Cytomation, Glostrup, Denmark; diluted 1:2000) overnight at 4 °C or with FITC conjugated TUNEL (C1088, Beyotime Biotechnology, Shanghai, China) for 1 h at 37 °C, using 1% BSA as a control, followed by incubation with Alexa Fluor 555-conjugated goat anti-rabbit antibody (A32732, Invitrogen, Waltham, MA, USA, diluted in 1:1000) for 2 h in the dark to visualize vWF. Sections were covered with Prolong Gold antifade reagent supplemented with DAPI (8961, Cell Signaling Technology, Danvers, MA, USA).

#### RNA-seq

Because all parameters studied on HUVEC proliferation, wound closure, tube formation and migration were significantly inhibited after treatment with 500 µM of ibuprofen and to exclude (1) a potential suboptimal (100 µM) and (2) a potential toxic (1000 µM) effect of ibuprofen on HUVEC we performed the RNA Seq experiments with 500 µM of ibuprofen. RNA-seq was performed on a BGIseq500 platform (BGI-Shenzhen, China). Briefly, isolated RNA from HUVECs treated with 0 or 500 µM ibuprofen was quantified using a NanoDrop and Agilent 2100 bioanalyzer (Thermo Fisher Scientific, MA, USA), and purified with Oligo(dT)-attached magnetic beads. After synthesis of the first-strand cDNA, A-tailing mix and RNA index adapters were added by incubating to end repair. The amplified cDNA fragments were purified by Ampure XP Beads and validated on the Agilent Technologies 2100 bioanalyzer for quality control. The double-stranded PCR products from the previous step were heated, denatured and circularized by the splint oligo sequence to get the final library. The single-strand circle DNA (ssCir DNA) was formatted as the final library. The final library was amplified with phi29 to make DNA nanoballs (DNB) which had more than 300 copies of one molecular. DNBs were loaded into the patterned nanoarray and single end 50 bases reads were generated on the BGIseq500 platform. The data was first filtered with SOAPnuke (v1.5.2, https://github.com/BGI-flexlab/SOAPnuke) to remove reads with sequencing adapter, high low-quality base ratio (> 20%), and unknown base ratio higher than 5%. Clean reads were mapped and the expression level was calculated by RSEM (V1.2.12, https://github.com/deweylab/RSEM). Differential expression analysis was performed using DESeq2 (v1.4.5). GO (http://www.geneontology.org/) and KEGG (https://www.kegg.jp/) enrichment analysis was performed to summarize the pathways involved. The significance was corrected by Q value with a rigorous threshold (Q value ≤ 0.05) by Bonferroni. The analysis was performed on the Dr. Tom platform generated by BGI (https://biosys.bgi.com/).

#### Cell cycle analysis and apoptosis by flow cytometry

For cell cycle analysis, HUVECs were fixed overnight with 70% ethanol at 4 °C after treatment with DMSO (0.1%, v/v) or ibuprofen for 48 h. Hereafter, HUVECs were incubated with Triton X100 (T8787, Sigma-Aldrich, St. Louis, MO, USA; 0.1%) and RNAse A (R-4875, Sigma-Aldrich, St. Louis, MO, USA; 100 µg/mL) and stained for 30 min with propidium iodide (PI, P4170, Sigma-Aldrich, St. Louis, MO, USA; 40 µg/mL). The fluorescence was excited at 488 nm and measured with a 585 nm filter. Data were analyzed with Modfit software (Verity Software House, Topsham, Maine, USA). For apoptosis analysis, HUVECs were treated with DMSO (0.1%, v/v) and ibuprofen for 72 h and stained with a FITC Annexin V Apoptosis Detection Kit I (556547, BD Life Sciences, Franklin Lakes, NJ, USA) according to the manufacturer’s instruction.

### Animal studies

All animal procedures in this study were approved by the Institutional Animal Care and Use Committee of the Shenzhen Institutes of Advanced Technology of the Chinese Academy of Sciences. Newborn pups were randomized into 4 groups (N = 6–7 for each group): two experimental BPD groups raised in 100% oxygen and 2 control groups raised in room air, assuming a similar sex ratio among the groups. Experimental BPD was induced by exposure to hyperoxia as previously reported [24]. Briefly, newborn pups were raised and fed by foster dams in a Plexiglas chamber filled with 100% oxygen for 10 days. Foster dams had access to water and food ad libitum and were rotated daily to prevent hyperoxia-induced lung damage and, importantly, balance maternal care given to pups in control and experimental groups. Pups received daily subcutaneous injections of 50 mg/kg of ibuprofen (I4883, Sigma-Aldrich, St. Louis, MO, USA), dissolved in 100 μL arginine buffer (10 mg/mL, SinePharm, Shanghai, China) or arginine buffer only as treatment control. Pups were anesthetized on day 10 by intraperitoneal injection of pentobarbital (40 mg/kg). Three independent experiments were performed, two for histological data and one for RT-PCR data. Lung tissue from pups raised in different litters was either fixed in situ with formalin or frozen in liquid nitrogen and stored at − 80 °C for RT-PCR as previously reported [24].

### Histology and lung morphometry

Formalin-fixed, paraffin-embedded, 4 µm-thick lung sections were stained with hematoxylin and eosin. In addition, lungs were stained with specific antibodies against von Willebrand factor (vWF, A0082, Dako Cytomation, Glostrup, Denmark; diluted 1:5000), CD31 (ab182981, Abcam, Fremont, CA, USA; diluted 1:2000), CD68 (monocytes and macrophages, ab31630, Abcam, Fremont, CA, USA; diluted 1:500), myeloperoxidase (MPO, ab208670, Abcam, Fremont, CA, USA; diluted 1:1000), α smooth muscle actin (ASMA, A2547, Sigma-Aldrich, St. Louis, MO, USA; diluted 1:10,000), or 1% BSA (A1933, Sigma-Aldrich, St. Louis, MO, USA) as a control, followed by staining with HRP conjugated anti-mice/rabbit antibodies accordingly (ab6721 or ab6728, Abcam, Fremont, CA, USA; diluted 1:1000). Antibody staining was visualized using the chromogenic substrate NovaRed as recommended by the manufacturer (SK-4800, Vector, Burlingame, CA, USA). Sections were counterstained briefly with hematoxylin using standard methods [24]. Mean linear intercept (MLI) was determined on HE stained lung sections, as previously reported [8, 24]. Briefly, 10 non-overlapping photos of lung tissues were made with an Olympus CX43 microscope (Tokyo, Japan) at a 200× magnification. Structures, including big vessels and airways, were excluded. The photos were analyzed using a coherent system of 21 lines and 42 points embedded in the CellSens software (Olympus, Tokyo, Japan).

Vessel density was assessed in lung sections stained for vWF or CD31 at a 200× magnification by counting the number of vessels per field. At least 10 representative fields per experimental animal were investigated. The density of ED-1 positive monocytes and macrophages or MPO-positive neutrophilic granulocytes was determined in the alveolar compartment by counting the number of cells per field. In each experimental animal 20 fields in one section were studied at a 400× magnification. Pulmonary alveolar septal thickness was assessed in HE-stained lung sections at a 400× magnification by averaging 100 measurements per 10 representative fields. Medial wall thickness was calculated from the formula “percent wall thickness = $\frac{2 \times wall \cdot thickness}{{external \cdot diameter}} \times 100$” [26]. Structures, including big vessels and airways, were excluded. Two independent researchers blinded to the experimental groups performed the analysis.

### Real-time RT-PCR

RNA was isolated from 30 mg lung tissue homogenates or 5 × 10^5^ HUVECs using TRIzol (#15596026, Invitrogen, Waltham, MA, USA) according to the supplier’s manual. cDNA was synthesized with the RevertAid First Strand cDNA Synthesis Kit (K1622, Thermo Scientific, Waltham, MA, USA). Real-time quantitative PCR was performed on an Applied Biosystems 7300 Plus system (Applied Biosystems, Foster City, CA, USA). β-actin was used as a housekeeping gene reference. Relative mRNA expression was normalized to room air controls. Primers are listed in Table 1.

**Table 1:** Sequences of oligonucleotides for forward and reverse primers for real-time RT-PCR

Gene product	Forward primer	Reverse primer
IL6	5′-ATATGTTCTCAGGGAGATCTTGGAA-3′	5′-TGCATCATCGCTGTTCATACAA-3′
CINC1	5′-GCACCCAAACCGAAGTCATA-3′	5′-GGGGACACCCTTTAGCATCT-3′
MCP1	5′-ATGCAGTTAATGCCCCAGTCA-3′	5′-TTCTCCAGCCGACTCATTGG-3′
TF	5′-CCCAGAAAGCATCACCAAGTG-3′	5′-TGCTCCACAATGATGAGTGTT-3′
β-Actin	5′-TTCAACACCCCAGCCATGT-3′	5′-AGTGGTACGACCAGAGGCATACA-3′

### Statistics

Parameters were displayed as mean ± standard error of the mean (SEM), unless otherwise stated. Differences between experimental groups were analyzed by one-way ANOVA, followed by Sidak multiple comparisons test. For comparison of survival curves, Kaplan–Meier analysis followed by a log rank test was performed. Statistical analysis was performed using a GraphPad Prism version 8 software package (San Diego, CA, USA). A* p*-value < 0.05 was considered statistically significant.

## Data availability

RNA sequencing data analyzed in the article (Fig. 7A) have been deposited into the CNGB Sequence Archive (CNSA) [27] of China National GeneBank DataBase (CNGBdb) [28] with accession number CNP 0002466.

## Results

### Effects of ibuprofen treatment on endothelial cell proliferation, migration and tube formation ability

**Fig. 1:**
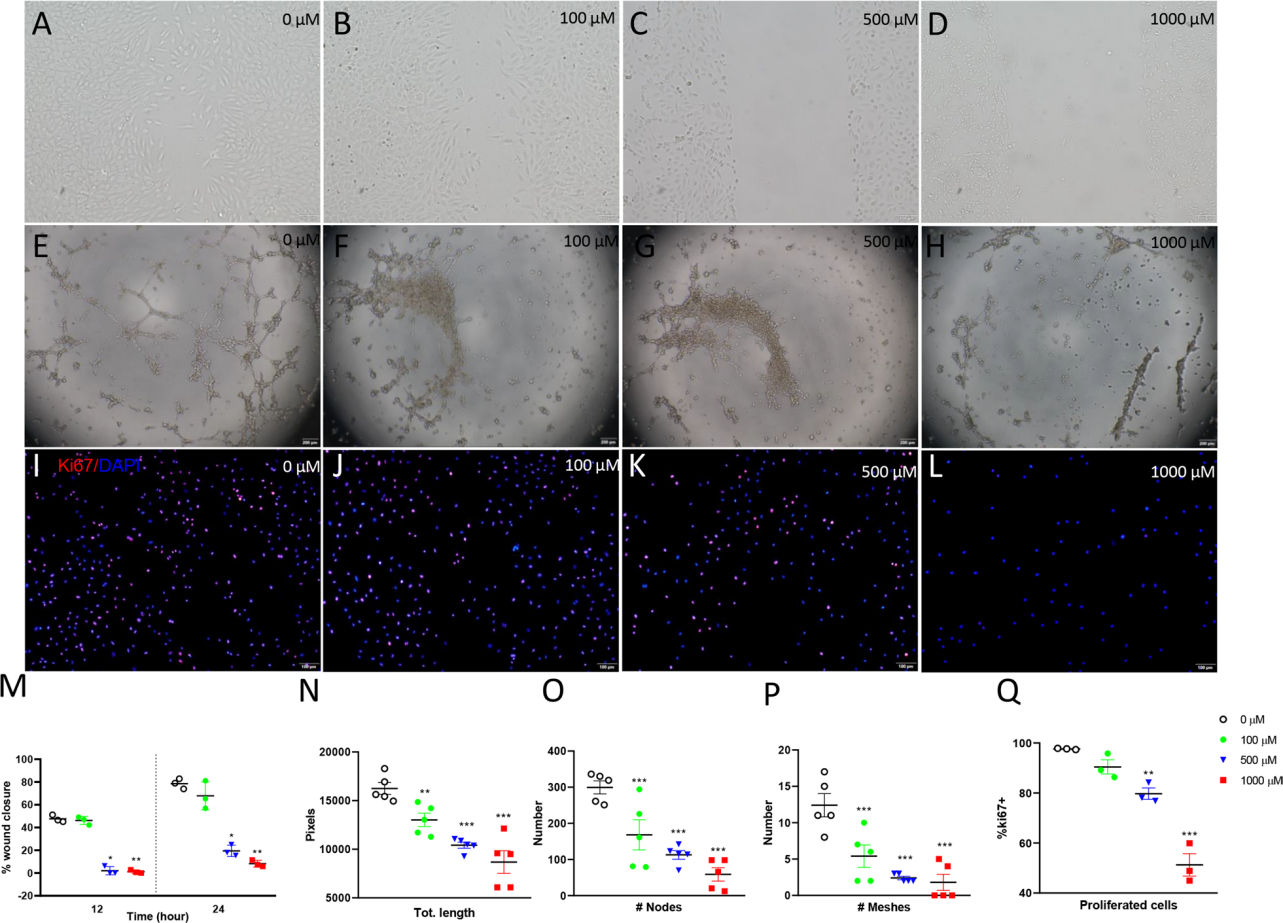
Representative photos of migration (**A**–**D**), tube formation (**E**–**H**) and proliferation (**I**–**L**) of HUVECs treated with DMSO (0.1%, v/v, **A**, **E**, **I**, open circles), 100 µM ibuprofen (**B**, **F**, **J**, green circles), 500 µM ibuprofen (**C**, **J**, **K**, blue triangles) and 1000 µM ibuprofen (**D**, **H**, **L**, red squares). Wound closure rate at 12 and 24 h after treatment (**M**, N = 3), total length, number of nodes and meshes (**N**–**P**, N = 5) and Ki67-positive cell ratio (**Q**, N = 3) were used to quantify the migration, tube formation and proliferation ability of HUVECs. Values are expressed as mean ± SD. *p < 0.05, **p < 0.01 and ***p < 0.001 versus DMSO controls

Since experimental evidence pointed to an inhibitory effect of ibuprofen on angiogenesis, we investigated the role of ibuprofen in endothelial function of human umbilical vein endothelial cells (HUVEC) in vitro by studying migration, neovascularization and proliferation. After 12 and 24 h of treatment with 500 or 1000 µM of ibuprofen, wound healing was attenuated by 4.0-fold (24 h; 500 µM; *p* < 0.05, Fig. 1C, M) or 9.6-fold (24 h; 1000 µM; *p* < 0.01, Fig. 1D, M). After 6 h of treatment with ibuprofen, tube formation ability was attenuated (Fig. 1E–H), as shown by reduced total length, number of nodes and number of meshes in HUVECs treated with 100, 500 or 1000 µM of ibuprofen (*p* < 0.01 and *p* < 0.001, Fig. 1N–P). After 24 h of treatment with 0, 100, 500 or 1000 µM of ibuprofen, cell proliferation was reduced by 1.2-fold (500 µM, *p* < 0.01, Fig. 1K, Q) and 1.9-fold (1000 µM, *p* < 0.001, Fig. 1L, Q), respectively.

### Ibuprofen arrested cell cycle at S stage in HUVEC

**Fig. 2:**
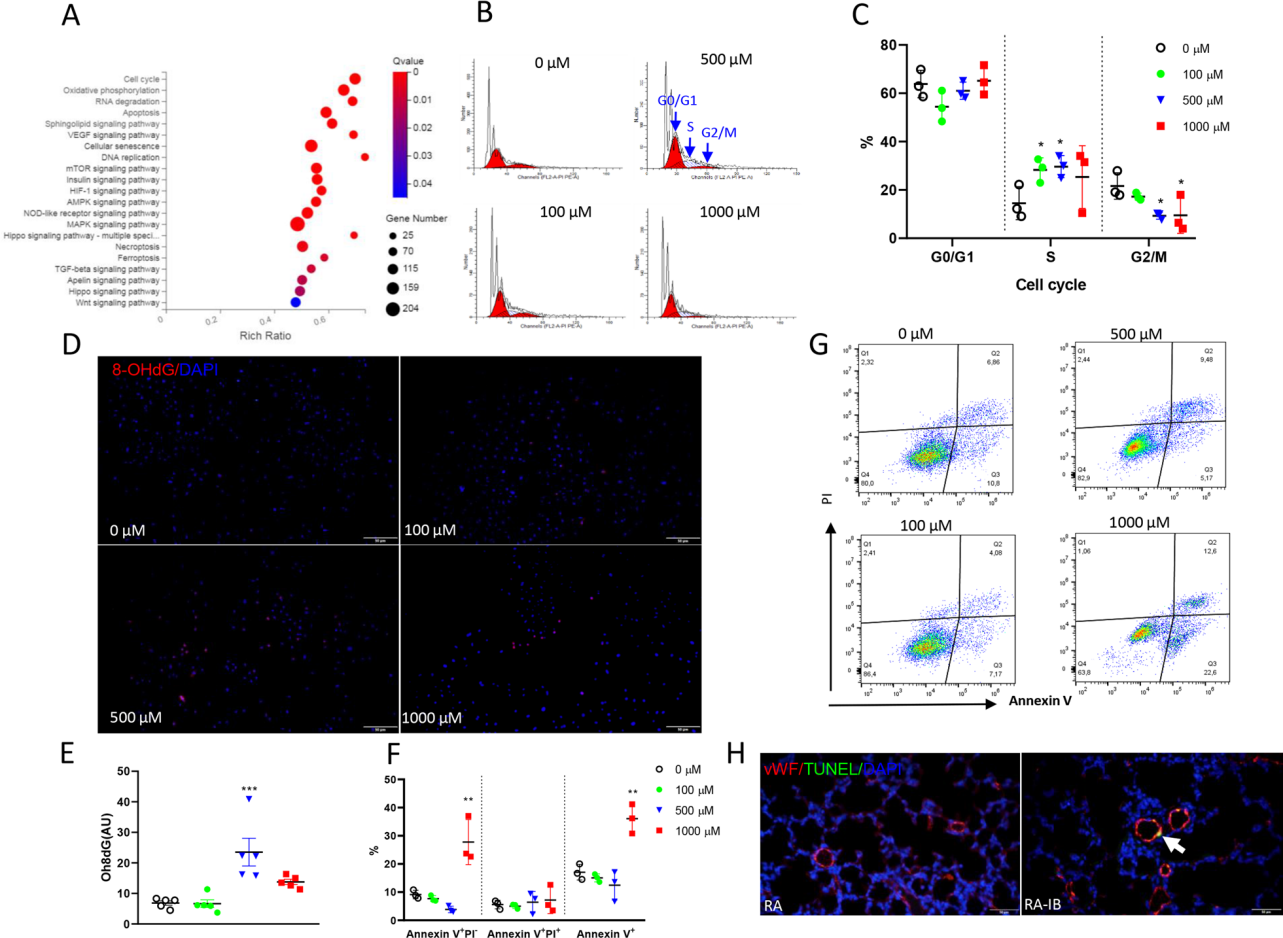
Bubble plot of pathways significantly affected by 500 µM of ibuprofen (**A**, N = 4–5). Representative photos of cell cycle analysis of HUVECs treated with DMSO (0.1%, v/v), 100, 500 or 1000 µM of ibuprofen (**B**) and proportion of cells at different cell cycle stages, G0/G1, S and G2/M (**C**, N = 3). Representative photos and fluorescence intensity quantification of the DNA damage marker, 8-hydroxy-2′-deoxyguanosine (8-OHdG) staining in HUVECs treated with DMSO or ibuprofen (**D** and **E**, N = 5). Representative photos and quantification of apoptosis analysis by propidium iodide (PI) and Annexin V staining in HUVECs treated with DMSO or ibuprofen (**G** and **F**, N = 3). Representative photos of TUNEL staining of neonatal rat lung tissue exposed to ibuprofen. Rat pups were kept in room air (RA) and were daily injected subcutaneously with arginine buffer (RA) or ibuprofen (50 mg/kg/day; RA-IB) until 10 days of age (**H**). DMSO, open circles; 100 µM ibuprofen, green circles; 500 µM ibuprofen, blue triangles; and 1000 µM ibuprofen, red squares. Values are expressed as mean ± SD (**C** and **F**) or mean ± SEM (**E**). *p < 0.05, **p < 0.01 and ***p < 0.001 versus DMSO controls

To further explore the effects of ibuprofen on HUVEC, RNA-seq was performed in control and 500 µM ibuprofen treated HUVECs. Several pathways in HUVECs were affected by ibuprofen, including VEGF signaling, apoptosis, oxidative phosphorylation and cell cycle regulation (Fig. 2A). Because the cell cycle was the most affected pathway, we studied this pathway in more detail in flow cytometry experiments and demonstrated that significantly more ibuprofen-treated HUVECs were in S phase and less cells in G2/M phase compared to controls, indicating that the cell cycle in HUVECs was arrested in S phase (Fig. 2B, C), when DNA was synthesized. Since DNA might be damaged by ibuprofen during the synthesis in S stage by oxidative stress [29], we studied the expression of the DNA damage marker, 8-hydroxy-2'-deoxyguanosine (8-OHdG). Increased expression of 8-OHdG indicated that ibuprofen induces oxidative stress in HUVECs, damages the DNA (Fig. 2D, E) and subsequently causes apoptosis (Fig. 2F, G). This was confirmed in neonatal rats treated with ibuprofen in which we observed more vWF-positive endothelial cells in lung tissue sections that were stained for TUNEL, a marker for the final phase of apoptosis (Fig. 2H).

### Effects of ibuprofen on growth and survival in neonatal rat pups

**Fig. 3:**
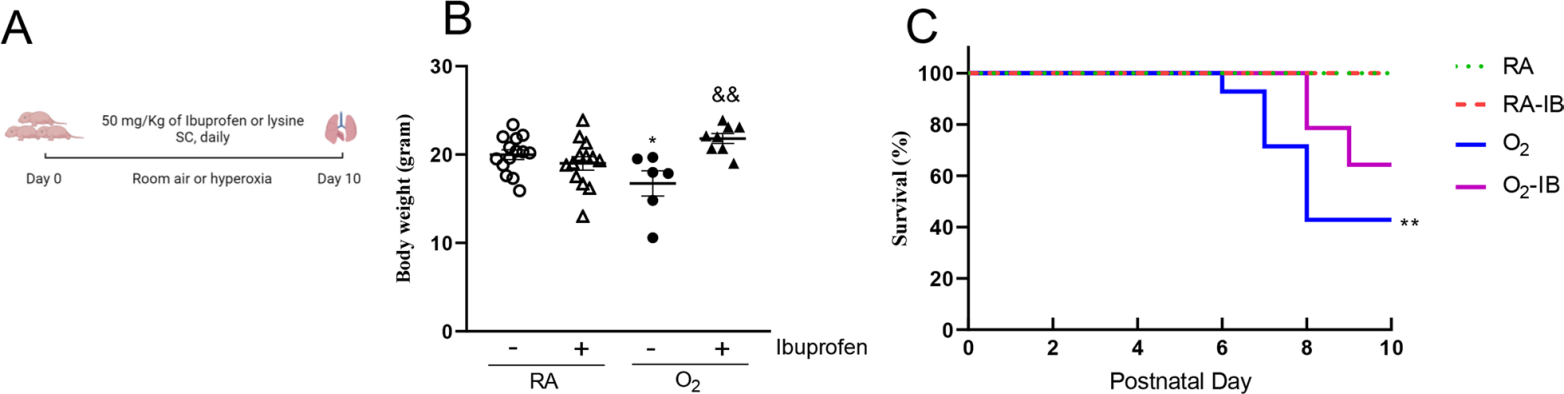
Experimental scheme (**A**), growth (**B**) on day 10 (N = 14 for the RA control group and N = 13 for the RA-ibuprofen group, N = 6 for the oxygen control group, and N = 8 for the oxygen-ibuprofen group) and Kaplan–Meier survival curve (**C**) (calculated from 14 pups for each group). Room air (RA) pups (open symbols) and age-matched O_2_-exposed (O_2_) pups (solid symbols) were injected daily with arginine buffer (circles) or ibuprofen (50 mg/kg/day; triangles) until 10 days of age. Data are expressed as mean ± SEM. *p < 0.05 and **p < 0.001 versus RA controls. ^&&^*p* < 0.05 versus age-matched arginine buffer-treated O_2_-exposed controls

Because vascular development and angiogenesis play a critical role in alveolarization during lung development and BPD pathogenesis these in vitro data in HUVECs prompted us to investigate the effect of ibuprofen on normal postnatal lung development and BPD pathogenesis in neonatal rats kept in normoxia (room air; RA) or in hyperoxia (100% O_2_), respectively. The experimental scheme is displayed in Fig. 3A. On neonatal day 10, body weight was comparable in arginine- and ibuprofen-treated rat pups kept in RA (19–20 g; Fig. 3B). Pups exposed to hyperoxia for 10 days showed a significant decrease in body weight (17 g), which was prevented by ibuprofen treatment (21 g). Exposure to hyperoxia resulted in a 43% survival on day 10 in arginine-treated rat pups (Fig. 3C). Treatment of experimental BPD with 50 mg/kg/day of ibuprofen resulted in a tendency towards less mortality (survival: 64%; Fig. 3C), which started 2 days later (Fig. 3C) compared to hyperoxia-exposed controls. All RA-exposed pups showed no morbidity or mortality during the experimental period of 10 days.

### Effects of ibuprofen on postnatal pulmonary vascular development

**Fig. 4:**
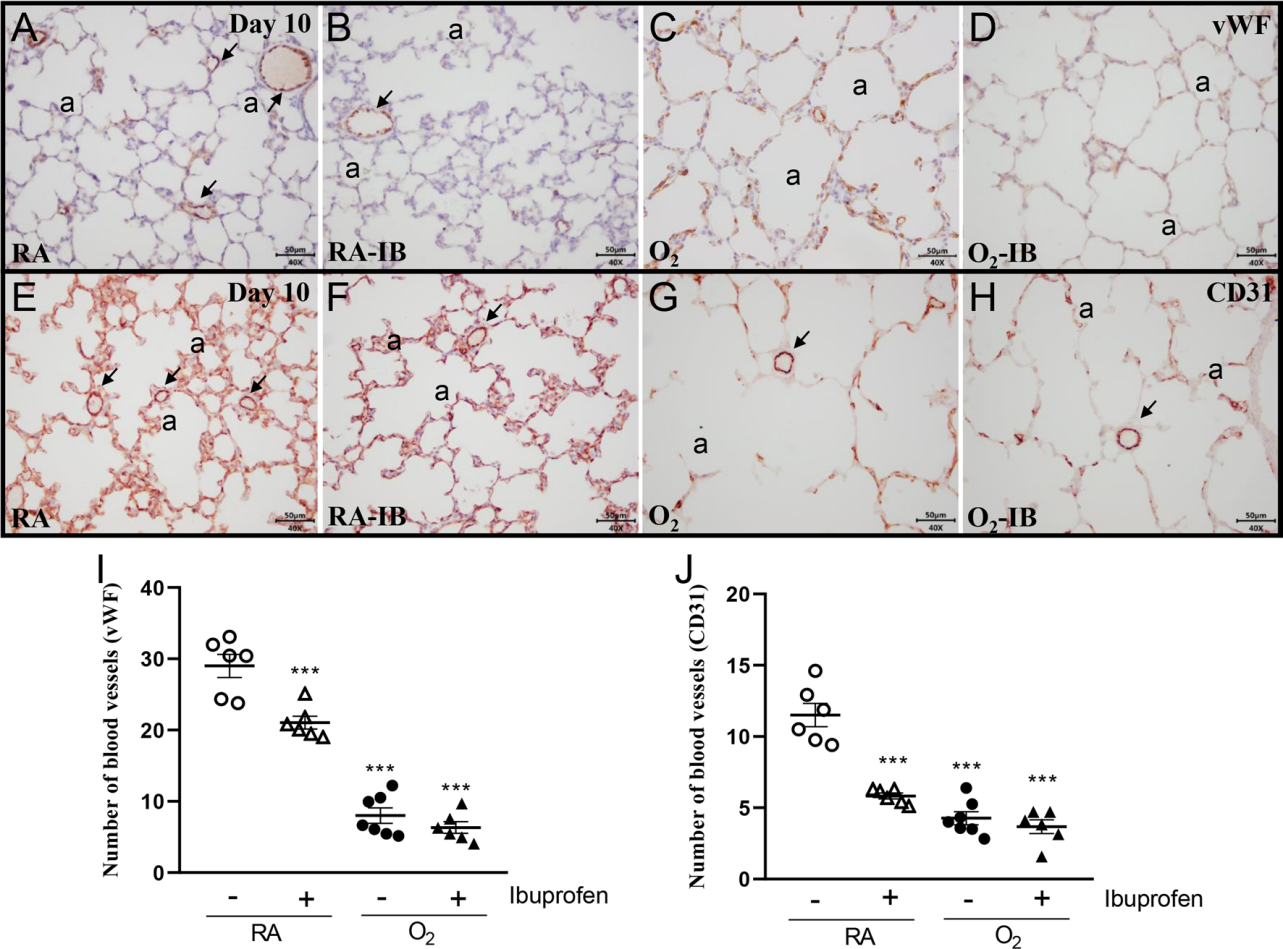
Representative lung sections stained for von Willebrand Factor (vWF; **A**–**D**) or CD31 (**E**–**H**) of rat pups kept in room air (RA; open symbols; **A**, **B**, **E** and **F**) or 100% O_2_ (solid symbols; **C**, **D**, **G** and **H**). Pups were injected daily with arginine buffer (circles; **A**, **C**, **E** and **G**) or ibuprofen (50 mg/kg/day; triangles; **B**, **D**, **F** and **H**) until 10 days of age. Arrows indicate blood vessels. The number of pulmonary vessels stained with vWF (**I**) or CD31 (**J**) was determined on paraffin sections in rat pups on day 10. Values are expressed as mean ± SEM (N = 6–7). ***p < 0.001 versus RA controls

Administration of ibuprofen to RA-exposed controls reduced blood vessel density: 1.6-fold for vWF staining (Fig. 4B, I) and 2.0-fold for CD31 staining (Fig. 4F, J), (*p* < 0.001). The anti-angiogenic effect of ibuprofen was less significant than exposure to hyperoxia, which dramatically reduced pulmonary vessel density (2.3-fold for vWF staining and 2.7-fold for CD31 staining, *p* < 0.001; Fig. 4C, G, I and J) compared to RA controls. In hyperoxia-exposed pups treated with ibuprofen, blood vessel density was not significantly different from hyperoxia-exposed treatment controls (Fig. 4D, H, I and J).

### Effects of ibuprofen on pulmonary inflammation and lung airway development

**Fig. 5:**
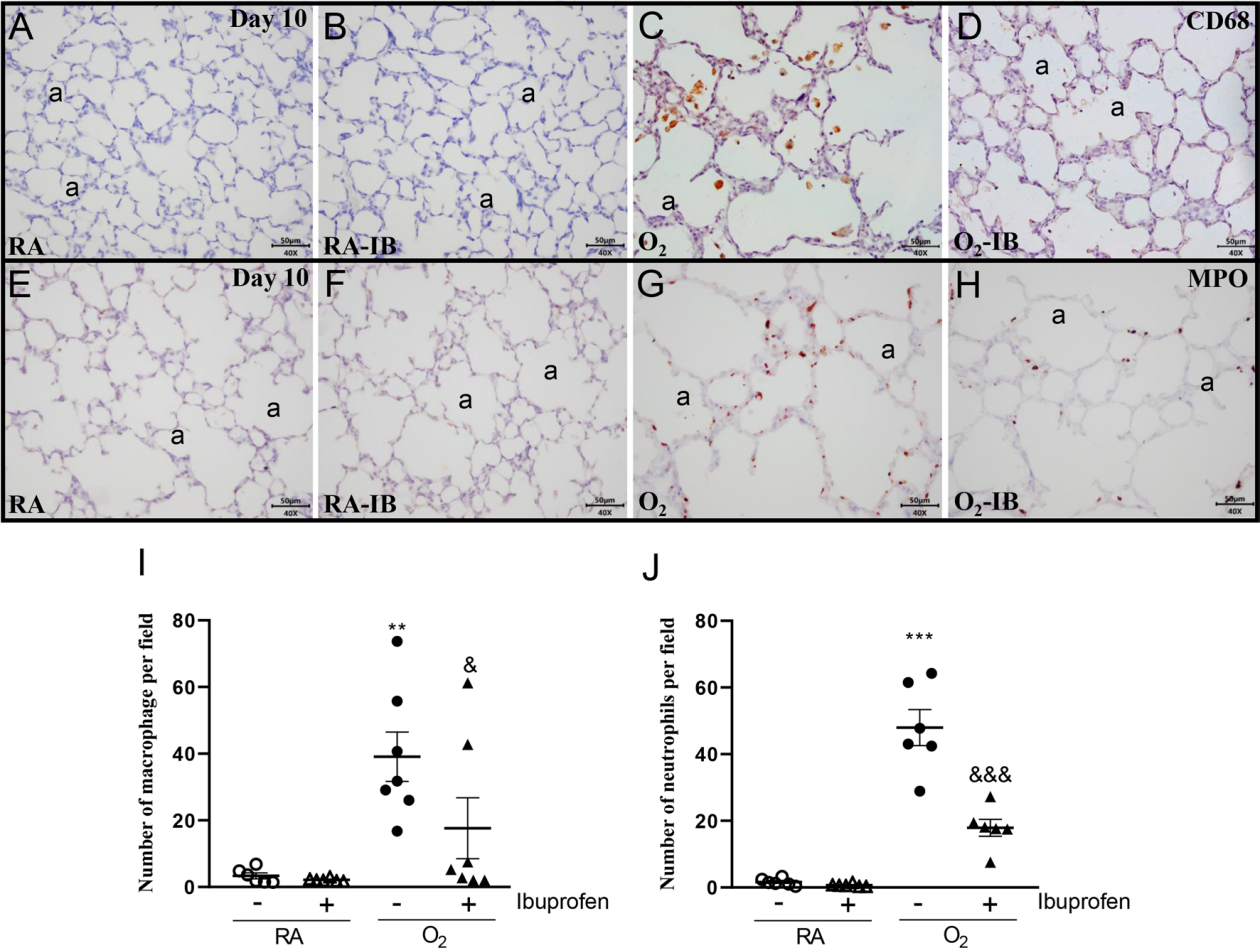
Representative lung sections stained for the macrophage marker CD68 (**A**–**D**) or myeloperoxidase (MPO) as a marker for neutrophilic granulocytes (**E**–**H**) of rat pups kept in room air (RA; **A**, **B**, **E** and **F**) or 100% O_2_ (**C**, **D**, **G** and **H**). Pups were injected daily with arginine buffer (**A**, **C**, **E** and **G**) or ibuprofen (50 mg/kg/day; **B**, **D**, **F** and **H**) until 10 days of age. a = alveolus. The influx of monocytes and macrophages (**I**) and neutrophilic granulocytes (**J**) was determined by morphometry on lung sections. RA pups (open symbols) and O_2_ pups (solid symbols) were injected daily with arginine buffer (circles) or ibuprofen (50 mg/kg/day; triangles) until 10 days of age. Values are expressed as mean ± SEM (N = 6–7). **p < 0.01 and ***p < 0.001 versus RA controls. ^&^*p* < 0.05 and ^&&&^*p* < 0.001 versus age-matched arginine buffer-treated O_2_-exposed controls

**Fig. 6:**
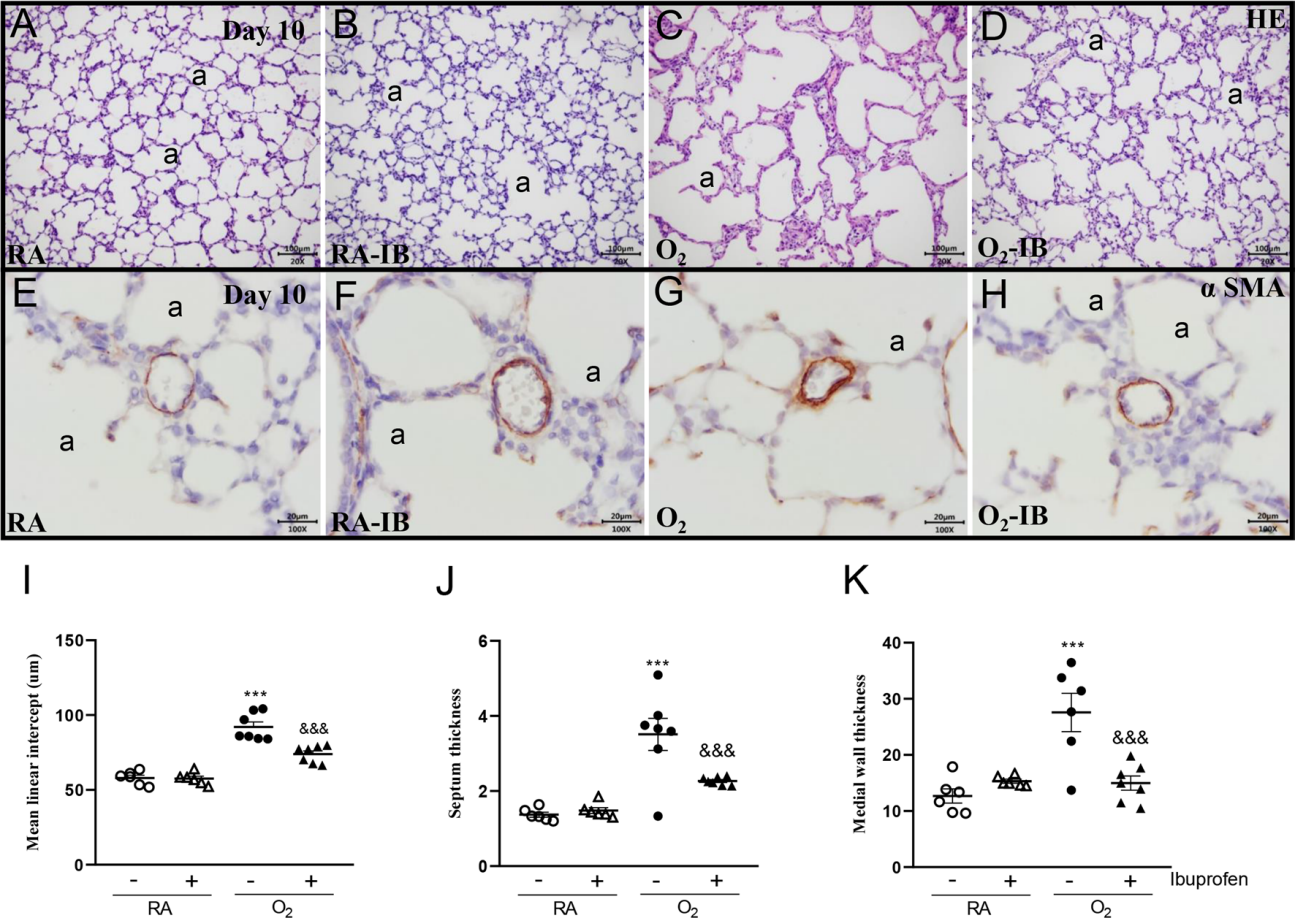
Representative lung sections stained for HE (**A**–**D**), or α smooth muscle actin (αSMA; **E**–**H**) of rat pups kept in room air (RA; **A**, **B**, **E** and **F**) or 100% O_2_ (**C**, **D**, **G** and **H**). Pups were injected daily with arginine buffer (**A**, **C**, **E** and **G**) or ibuprofen (50 mg/kg/day: **B**, **D**, **F** and **H**) until 10 days of age. a = alveolus. Lung morphometry, including the quantification of mean linear intercept (MLI, **I**), septal thickness (**J**), and arterial medial wall thickness (**K**), was determined on paraffin sections from rat pups on day 10. RA pups (open symbols) and O_2_ pups (solid symbols) were injected daily with arginine buffer (circles) or ibuprofen (50 mg/kg/day; triangles) until 10 days of age. Values are expressed as mean ± SEM (N = 6–7). ***p < 0.001versus RA controls. ^&&^*p* < 0.01 and ^&&&^*p* < 0.001 versus age-matched arginine buffer-treated O_2_-exposed controls

Since ibuprofen is a potent anti-inflammation drug, we studied the effect of ibuprofen on pulmonary inflammation induced by hyperoxia. Oxygen exposure for 10 days resulted in an influx of macrophages (11.8-fold, *p* < 0.01 Fig. 5C, I) and neutrophils (29.2-fold, *p* < 0.001, Fig. 5G, J). Administration of 50 mg/kg/day of ibuprofen to hyperoxia-exposed pups significantly reduced the influx of macrophages (2.2-fold, p < 0.05; Fig. 5D, I) and neutrophils (2.7-fold, p < 0.001; Fig. 5H, J). Because inflammation and vascular development are key mediators in BPD, we investigated the effect of ibuprofen on lung airway development. Hyperoxia led to a heterogeneous distribution of enlarged air-spaces (1.6-fold, *p* < 0.001; Fig. 6C, I), surrounded by septa with increased thickness (2.6-fold, *p* < 0.001; Fig. 6C, J), and increased pulmonary arterial wall thickness (2.2-fold, *p* < 0.001; Fig. 6G, K). Compared to hyperoxia exposed controls, ibuprofen reduced alveolar size (1.3-fold, *p* < 0.001; Fig. 6D, I), alveolar septal thickness (1.5-fold, *p* < 0.001; Fig. 6D, J), and pulmonary arterial wall thickness (1.8-fold, *p* < 0.001; Fig. 6H, K). Administration of ibuprofen to RA-exposed controls did not affect the parameters investigated.

### Effects of ibuprofen treatment on mRNA expression in lung tissue of genes involved in inflammation and coagulation.

**Fig. 7:**
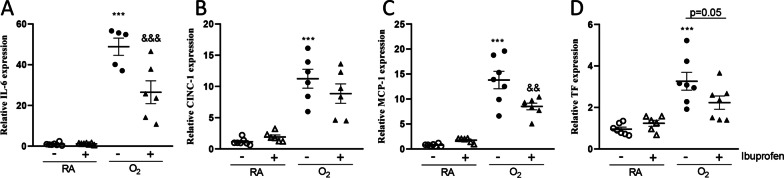
Relative mRNA expression in lung homogenates of Interleukin 6 (IL6; **A**), chemokine-induced neutrophilic chemoattractant-1 (CINC-1; **B**), monocyte chemoattractant protein 1 (MCP1; **C**) and tissue factor (TF; **D**) on day 10 in rat pups. RA pups (open symbols) and O_2_ pups (solid symbols) were injected daily with arginine buffer (circles) or ibuprofen (50 mg/kg/day; triangles) until 10 days of age. Values are expressed as mean ± SEM (N = 5–7). ***p < 0.001 versus RA controls. ^&&^*p* < 0.01, ^&&&^*p* < 0.001 versus age-matched arginine buffer-treated O_2_-exposed controls

To explore the anti-inflammatory effect of ibuprofen in experimental BPD, presented in Fig. 7, we studied mRNA expression of key genes involved in inflammation and coagulation. Exposure of neonatal rat pups to hyperoxia for 10 days increased mRNA expression in the lung of the inflammatory factors interleukin 6 (IL-6, 49.7-fold, *p* < 0.001; Fig. 7A), chemokine-induced neutrophilic chemoattractant-1 (CINC-1, 9.8-fold, *p* < 0.001; Fig. 7B), and monocyte chemoattractant protein 1 (MCP-1, 13.8-fold, *p* < 0.001; Fig. 7C), the pro-coagulant factor tissue factor (TF, 3.3-fold, *p* < 0.001; Fig. 7D), compared to RA controls. Administration of ibuprofen for 10 days to hyperoxia-exposed rat pups significantly reduced mRNA expression of IL-6, MCP-1 and TF (*p* < 0.001, *p* < 0.01 and *p* = 0.05, respectively, Fig. 7A, C and D) compared to hyperoxia controls.

## Discussion

Ibuprofen compromised endothelial function in HUVECs by inducing oxidative stress-related DNA damage and arresting the cell cycle in the S-phase, which subsequently promoted endothelial apoptosis. The anti-angiogenic effect of ibuprofen in HUVECs, demonstrated by inhibition of cell proliferation, migration and tube formation ability, confirms previously published data [30]. Because ibuprofen is frequently used to treat a patent ductus arteriosus after premature birth, a patient population at risk of developing BPD, and angiogenesis plays a crucial role in normal and aberrant postnatal lung development, these findings prompted us to study the role of ibuprofen in normal neonatal lung development and in the pathogenesis of experimental BPD in rats [18, 31]. The anti-angiogenic effect of ibuprofen in HUVECs was confirmed in vivo in rat pups in which ibuprofen treatment during normal neonatal lung development had adverse effects on pulmonary vascular development that resulted in a reduced vascular bed. However, beneficial effects were also demonstrated in rat pups with hyperoxia-induced experimental BPD in which treatment with ibuprofen attenuated disease progression and lung injury by reducing lung inflammation, preventing pulmonary vascular remodeling and preserving alveolar development.

Ibuprofen-induced inhibition of angiogenesis was demonstrated by a reduced vascular bed in newborn rat pups raised in normoxia showing a reduced number of blood vessels after ibuprofen treatment using two different endothelial markers: vWF and CD31. An ibuprofen-induced inhibition of vascular development was observed in rat ocular development [21], cardiovascular development in zebrafish [22] and tumor growth and metastasis [30, 32]. The potential mechanisms involved include inhibition of vascular growth factors, like vascular endothelial growth factor (VEGF), fibroblast growth factor (FGF), and hypoxia-inducible factors (HIF) [33–35], inhibition of the mitogen-activated protein (MAP) kinase (ERK2) activity [36], and direct inhibition of cell function [32]. Here, we found that multiple pathways were affected in ibuprofen-treated HUVECs, including the well-known angiogenesis related pathways: VEGF signaling, HIF signaling and Hippo signaling, as well as processes involved in cell cycle, apoptosis, senescence, necroptosis and ferroptosis, of which the cell cycle was the most significantly affected one. We further confirmed the arrest of the cell cycle at S stage in ibuprofen treated HUVECs. Although much is known about the regulation of the G1/S, G2/M, and metaphase/anaphase transitions by different cyclin-dependent kinases (cDKs) and their activating cyclin subunits, less is known about the control mechanism for the S/G2 transition. The expression of CDK1/2, CHK1 and cyclin A, which were suggested to be involved in S phase or S/G2 transition [37], were significantly down regulated by ibuprofen in the RNA-seq data (data not shown). In addition, experimental evidence strongly suggests that DNA damage is a trigger for S/G2 arrestment [38]. Therefore, we examined the 8-OHdG level to indicate DNA injury and found that ibuprofen increased 8-OHdG expression, probably reflecting DNA damage in HUVECs, thereby leading to the arrest in the S stage and resulting in cell apoptosis shown in this study and by others [29, 34, 39]. Besides, we also found the apelin/APJ pathway as one of the most affected pathways by ibuprofen in our RNA seq data (Fig. 2A), in which apelin is a potent vasodilator and protects effectively against experimental BPD in rat pups [9].

Inflammation plays a pivotal role in the pathogenesis of BPD and may contribute to severe lung tissue damage and fibrosis, and treatment with anti-inflammatory agents protects against hyperoxia-induced experimental BPD [11, 40, 41]. Since ibuprofen is a potent nonsteroidal anti-inflammatory drug, we expected an anti-inflammation effect of ibuprofen in our experimental model. Indeed, ibuprofen protected against hyperoxia-induced lung injury in rat pups by reducing the influx of inflammatory cells, mRNA expression of pro-inflammatory genes, vascular remodeling and alveolar enlargement in the current study. The anti-inflammatory effect of ibuprofen in neonatal rats with experimental BPD is supported by observations in multiple in vivo models of lung disease in dogs, rabbits and sheep with sepsis, in mice with trauma and septic challenge, in rats with ventilator-induced or endotoxic lung injury, in sheep with thrombin-induced lung vascular leakage [42–44] and in cystic fibrosis patients with lung inflammation [45]. The mechanism of anti-inflammation by ibuprofen has been established by blocking COXs activity, thereby attenuating prostaglandin mediated inflammation [46]. Our data confirm previous studies demonstrating protection against hyperoxia-induced BPD in rodents treated with (selective) COX2 inhibitors, including aspirin and celecoxib and in genetically modified COX2^−/−^mice [47].

The absence of alveolar enlargement in ibuprofen treated rat pups kept in normoxia and the beneficial effect of ibuprofen on aberrant alveolar development and vascular remodeling in experimental BPD was unexpected, because in BPD pathogenesis alveolar enlargement is believed to be driven by aberrant vascular development [1, 31]. We speculate that (1) despite its anti-angiogenic effect ibuprofen preserves vascular integrity, thereby preventing alveolar enlargement in rat pups kept in normoxia and (2) ibuprofen alleviates BPD pathology in rat pups kept in hyperoxia by reducing the inflammatory response and preserving vascular integrity thereby preventing aberrant alveolar development and vascular remodelling. Our findings are supported by experimental data by Kuniyoshi et al. [48], who found reduced alveolarization in neonatal rats treated with indomethacin, but not in ibuprofen treated neonatal rats. Their histological data clearly demonstrate that, in contrast to indomethacin treatment, early and late treatment with ibuprofen prevents alveolar enlargement in neonatal rats with experimental BPD. However, this beneficial effect of ibuprofen on alveolar enlargement in experimental BPD was not claimed by Kuniyoshi et al. [48]. A protective effect of ibuprofen in the lung was also demonstrated in premature baboons on lung development and in adult rats with ventilator-induced lung injury [43, 49].

Although clinical studies suggest that ibuprofen treatment for PDA closure in very premature infants might be a risk factor for PAH [19], our data do not support this potential adverse effect. We demonstrated that treatment of neonatal rats with ibuprofen had no adverse effects on arterial vascular remodeling during normal postnatal development and even prevented vascular remodeling in pups with experimental BPD, which is a readout for PAH in this experimental BPD model [11, 50]. The beneficial effects of ibuprofen on pulmonary vascular remodeling were unexpected, because reduced intracellular cAMP levels caused by prostaglandin inhibition in vascular smooth muscle cells are expected to exacerbate PAH [51, 52]. This unexpected finding may be explained indirectly via a dampening of the inflammatory response by ibuprofen, thereby preserving endothelial cell integrity and function, and reducing smooth muscle cell proliferation and contraction [53]. Alternatively, the beneficial effect on vascular remodeling can also be mediated via ibuprofen’s off-target effect of elevating intracellular cGMP levels via cGMP-selective phosphodiesterase (PDE) inhibition [54, 55]. This explanation is supported by the beneficial effects of agents that increase intracellular cGMP levels, either by stimulating the NO-eNOS-cGMP pathway with inhaled NO, apelin or soluble guanylate cyclase modulators or inhibiting cGMP breakdown with the specific cGMP-selective PDE5 inhibitor sildenafil, in newborn rats with experimental BPD that our group and others described previously [9, 50, 56–58]. Interestingly, the beneficial effects of ibuprofen on experimental BPD may be explained by activation of the apelin/APJ pathway, which we have demonstrated to protect against experimental BPD in rats [9].

In this study, we exposed pups to ibuprofen for the whole experimental period (10 days), which varies from clinical practice, where ibuprofen is usually given to preterm infants for 3 days to close a PDA. Short versus prolonged and early versus late exposure to ibuprofen may affect its anti-inflammatory and anti-angiogenic effects. The anti-inflammatory effect of ibuprofen might be absent if ibuprofen is given for a short period and inflammation has not yet been established. Similarly, the anti-angiogenic effect of ibuprofen might be absent if given at a later stage when vascular growth is less vulnerable. We have recently demonstrated that ibuprofen reduces vascular growth factors, such as PDGF-BB, VEGF-A and HIF-2ɑ, in infants with PDA [59], confirming that the anti-angiogenic effect of ibuprofen is already present in human infants exposed for 3 days. Although this adverse effect on vascular growth might be absent when ibuprofen is given at a later stage, it may compromise its positive effect on PDA closure. This is in line with a recent clinical trial showing that ibuprofen significantly increased the risk of BPD in infants with a PDA [60] in the absence of its anti-inflammatory and presence of its anti-angiogenic effect. In the experimental BPD pups both the anti-angiogenic and anti-inflammatory effects were present and this might explain the different findings between our and clinical studies.

We acknowledge several limitations in this work. We used HUVECs in the in vitro experiments to study the effects of ibuprofen on angiogenesis. Although HUVECs are primary endothelial cells isolated from the umbilical cord vein and widely used in endothelial function studies, there might be fundamental differences between pulmonary micro vessels and the umbilical cord vein. Furthermore, since the ductus arteriosus closes naturally within 3 days in newborn rodent pups, we could not investigate the influence of ibuprofen on ductus closure, and the associated effect on BPD conferred by our and other clinical studies. Furthermore, we did not determine the gender of the pups in our study, obliviating the possibility to establish a potential difference in ibuprofen effect between males and females.

Ibuprofen exhibits an anti-angiogenic effect in HUVECs and the developing lung, which is considered an adverse effect in lung development and the pathogenesis of BPD, and beneficial effects in experimental BPD by promoting alveolarization, reducing inflammation and preventing vascular remodeling*.* This suggests that the beneficial effects of ibuprofen outperform the adverse effects in hyperoxia-induced experimental BPD in rat pups. However, extrapolation of the beneficial effects of ibuprofen and other NSAIDs to premature infants with BPD should be done with extreme caution. Similarly, prolonged and repeated courses of ibuprofen treatment for PDA closure in premature infants should be carefully considered.

## Data Availability

The datasets used and/or analyzed in this study are available from the corresponding author on reasonable request.

## Abbreviations

ACTB: Beta-actin
ASMA: α Smooth muscle actin
BPD: Bronchopulmonary dysplasia
CINC1: Chemokine-induced neutrophilic chemoattractant-1
CLD: Chronic lung disease
ECM: Endothelial cell medium
HUVECs: Human umbilical vein endothelial cells
IL-6: Interleukin 6
MLI: Mean linear intercept
MCP1: Monocyte chemoattractant protein 1
MPO: Myeloperoxidase
NSAID: Nonsteroidal anti-inflammatory drug
8-OHdG: 8-Hydroxy-2′-deoxyguanosine
PAH: Pulmonary arterial hypertension
PDA: Patent ductus arteriosus
TF: Tissue factor
TUNEL: Terminal deoxynucleotidyl transferase dUTP nick end labeling
VEGF: Vascular endothelial growth factor
vWF: Von Willebrand factor
